# Anatomical considerations and clinical interpretation of the 12-lead ECG in the prone position: a prospective multicentre study

**DOI:** 10.1093/europace/euac099

**Published:** 2022-10-05

**Authors:** Jorge Romero, Mario Garcia, Juan Carlos Diaz, Mohamed Gabr, Joan Rodriguez-Taveras, Eric D Braunstein, Sutopa Purkayastha, Maria T Gamero, Isabella Alviz, Jorge Marín, Julián Aristizábal, Olga Reynbakh, Adelqui O Peralta, Mauricio Duque, Kartikeya P Dave, Daniel Rodriguez, Cesar Nino, David Briceno, Alejandro Velasco, Kevin Ferrick, Leandro Slipczuk, Andrea Natale, Luigi Di Biase

**Affiliations:** Cardiac Arrhythmia Center, Division of Cardiology, Department of Medicine, Montefiore-Einstein Center for Heart and Vascular Care, Albert Einstein College of Medicine, 111 E 210th street, Bronx, NY 10467, USA; Cardiac Arrhythmia Center, Division of Cardiology, Department of Medicine, Montefiore-Einstein Center for Heart and Vascular Care, Albert Einstein College of Medicine, 111 E 210th street, Bronx, NY 10467, USA; Cardiology Department, Clínica Las Americas, Medellín, Colombia; Cardiac Arrhythmia Center, Division of Cardiology, Department of Medicine, Montefiore-Einstein Center for Heart and Vascular Care, Albert Einstein College of Medicine, 111 E 210th street, Bronx, NY 10467, USA; Cardiac Arrhythmia Center, Division of Cardiology, Department of Medicine, Montefiore-Einstein Center for Heart and Vascular Care, Albert Einstein College of Medicine, 111 E 210th street, Bronx, NY 10467, USA; Cardiac Arrhythmia Center, Division of Cardiology, Department of Medicine, Montefiore-Einstein Center for Heart and Vascular Care, Albert Einstein College of Medicine, 111 E 210th street, Bronx, NY 10467, USA; Cardiac Arrhythmia Center, Division of Cardiology, Department of Medicine, Montefiore-Einstein Center for Heart and Vascular Care, Albert Einstein College of Medicine, 111 E 210th street, Bronx, NY 10467, USA; Cardiac Arrhythmia Center, Division of Cardiology, Department of Medicine, Montefiore-Einstein Center for Heart and Vascular Care, Albert Einstein College of Medicine, 111 E 210th street, Bronx, NY 10467, USA; Cardiac Arrhythmia Center, Division of Cardiology, Department of Medicine, Montefiore-Einstein Center for Heart and Vascular Care, Albert Einstein College of Medicine, 111 E 210th street, Bronx, NY 10467, USA; Cardiology Department, Clínica Las Americas, Medellín, Colombia; Cardiology Department, Clínica Las Americas, Medellín, Colombia; Cardiac Arrhythmia Center, Division of Cardiology, Department of Medicine, Montefiore-Einstein Center for Heart and Vascular Care, Albert Einstein College of Medicine, 111 E 210th street, Bronx, NY 10467, USA; VA Boston Healthcare System, Division of Cardiology, Harvard Medical School, Boston, MA, USA; Cardiology Department, Universidad CES, Medellín, Colombia; Cardiac Arrhythmia Center, Division of Cardiology, Department of Medicine, Montefiore-Einstein Center for Heart and Vascular Care, Albert Einstein College of Medicine, 111 E 210th street, Bronx, NY 10467, USA; Cardiac Arrhythmia Center, Division of Cardiology, Department of Medicine, Montefiore-Einstein Center for Heart and Vascular Care, Albert Einstein College of Medicine, 111 E 210th street, Bronx, NY 10467, USA; Cardiology Department, Clínica Las Americas, Medellín, Colombia; Cardiac Arrhythmia Center, Division of Cardiology, Department of Medicine, Montefiore-Einstein Center for Heart and Vascular Care, Albert Einstein College of Medicine, 111 E 210th street, Bronx, NY 10467, USA; Cardiac Arrhythmia Center, Division of Cardiology, Department of Medicine, Montefiore-Einstein Center for Heart and Vascular Care, Albert Einstein College of Medicine, 111 E 210th street, Bronx, NY 10467, USA; Cardiac Arrhythmia Center, Division of Cardiology, Department of Medicine, Montefiore-Einstein Center for Heart and Vascular Care, Albert Einstein College of Medicine, 111 E 210th street, Bronx, NY 10467, USA; Cardiac Arrhythmia Center, Division of Cardiology, Department of Medicine, Montefiore-Einstein Center for Heart and Vascular Care, Albert Einstein College of Medicine, 111 E 210th street, Bronx, NY 10467, USA; Texas Cardiac Arrhythmia Institute, St David’s Medical Center, Austin, Texas, USA; Cardiac Arrhythmia Center, Division of Cardiology, Department of Medicine, Montefiore-Einstein Center for Heart and Vascular Care, Albert Einstein College of Medicine, 111 E 210th street, Bronx, NY 10467, USA

**Keywords:** Electrocardiogram, Prone position, Acute respiratory distress syndrome, 12-Lead ECG, Electrocardiography

## Abstract

**Aims:**

The aim of this study is to provide guidance for the clinical interpretation of electrocardiograms (ECGs) in prone position and to establish the electroanatomic explanations for the possible differences to supine position ECGs that may be observed. Additionally, to determine if prone back ECG can be used as an alternative to standard ECG in patients who may benefit from prone position.

**Methods and results:**

The ECG in supine (standard ECG), prone back (precordial leads placed on the patient’s back), and prone anterior position (precordial leads placed in the standard position with the subjects in prone position) were prospectively examined on 85 subjects. Comparisons of ECG parameters between these positions were performed. Computed tomography (CT) scans were performed in both positions to determine possible electroanatomic aetiologies for prone-associated ECG changes. There were significant differences in QRS amplitude in Leads V1–V5 between supine and prone positions. Q waves were more frequently observed in prone back position vs. supine position (V1: 74.1 vs. 10.6%, *P* < 0.0001; V2: 23.5 vs. 0%, *P* < 0.0001, respectively). Flat and inverted T waves were more common in prone back leads (V1: 98 vs. 66%, *P* < 0.0001; V2: 96 vs. 8%, *P* < 0.0001; V3: 45 vs. 7%, *P* < 0.0001). The 3D-CT reconstructions measurements corroborated the significant inverse correlation between QRS amplitude and the distance from the centre of the heart to the estimated lead positions.

**Conclusion:**

In prone back position ECG, low QRS amplitude should not be misinterpreted as low voltage conditions, neither should Q waves and abnormal T waves are considered anteroseptal myocardial infarction. These changes can be explained by an increased impedance (due to interposing lung tissue) and by the increased distance between the electrodes to the centre of the heart.

## Introduction

Prone position has been utilized over the last decades during surgeries (particularly those involving the neck, posterior head, spine, and retroperitoneal space), as well as adjunctive therapy to mechanical ventilation in patients with acute respiratory distress syndrome (ARDS). Furthermore, even in patients with ARDS who are on supplemental oxygen or non-invasive ventilation, prone position may improve oxygenation and reduce mortality.^1^ These effects are attributed to improved ventilation/perfusion matching, recruitment of dorsal lung regions, changes in lung compliance, and decreased overdistension in non-dependent lung regions.^2^

**What’s new?**

- 3D-CT images were used to corroborate that there is a significant inverse correlation between the distance from the centre of the heart to the electrodes’ expected location and the QRS amplitude.
- The changes observed in prone back ECG can be explained by an increased impedance and the ventral and caudal shift observed in the heart during prone position.
- This study enhanced a more accurate interpretation of the prone back ECG.

Conditions causing ARDS are frequently associated with cardiac complications, including myocardial infarctions, arrhythmias, and myocarditis.^3–5^ However, as electrocardiogram (ECG) standardization criteria have been designed only for patients in the supine position,^6^ placement of posterior precordial leads for prone back ECG (pbECG) can be challenging, and its correct interpretation could directly affect clinical decision-making. Consequently, patients who are placed in prone position are usually repositioned to supine position and back to prone position during ECG acquisition, a process that requires at least 3–5 skilled healthcare workers and can be tedious and time-consuming. In addition, this process can result in significant haemodynamic and respiratory instability, accidental endotracheal tube dislodgement,^7^ or in case of patients undergoing spinal surgery, be technically impossible. Therefore, we sought to assess the feasibility of accurately interpreting prone back position ECGs and to compare their findings among healthy volunteers and patients hospitalized with ARDS.

## Methods

### Study design and participants

This prospective study included healthy volunteers and patients hospitalized with ARDS at two academic institutions (Montefiore-Einstein Center for Heart and Vascular Care, Albert Einstein College of Medicine, Bronx; and Clinica Las Americas, Medellin, Colombia). Twelve-lead pbECG position (precordial leads placed on the patient’s back) and supine (standard ECG) were acquired for the entire cohort. In addition, prone anterior ECGs (paECG; precordial leads placed in the standard position with the subjects in prone position) were obtained in the healthy volunteer cohort. Subjects with intraventricular conduction abnormalities previous history of myocardial infarction, left ventricle hypertrophy, or an electrolyte abnormality were excluded from the study. The study was approved by the institutional review board at both institutions, and all patients and healthy volunteers gave written informed consent to participate in the study. In case of altered mental status or unconsciousness, written informed consent was asked from the patient’s proxy.

### Electrocardiograms and lead placement

**Figure 1:**
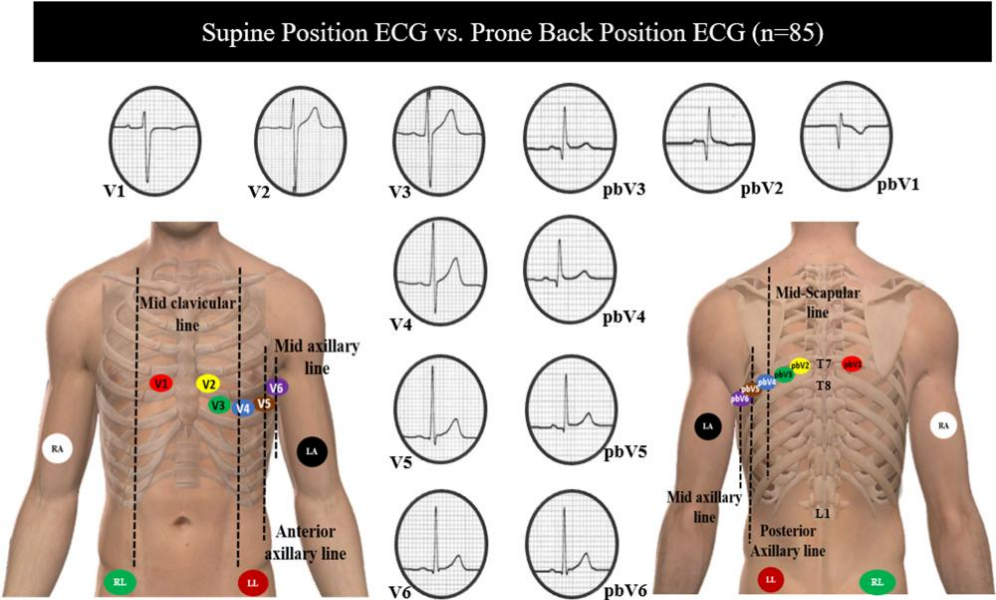
Central illustration: supine and prone back electrocardiogram lead location.

Electrocardiograms were obtained using the GE MAC 5500ECG machines (GE Healthcare, Chicago, IL, USA). For all subjects, limb electrodes were placed in the standard right arm (RA), left arm (LA), left leg (LL), and right leg (RL) positions. In pbECG, the precordial leads were placed as follows: pbV1 in the right paraspinal region at the level of the T7 vertebra, pbV2 in the left paraspinal region at the level of the T7 vertebra, pbV4 in the mid-scapular region at the level of the T8 vertebra (approximately below the tip of the scapula), pbV3 halfway between pbV2 and pbV4, pbV5 at the posterior axillary line at the level of the T8 vertebra, and pbV6 at the mid-axillary line at the level of T8 vertebra (*Figure 1*)

In the supine position, 12-lead ECG standard precordial lead placement was used (i.e. V1 in the fourth intercostal space at the right sternal border, V2 in the fourth intercostal space at the left sternal border, V4 in the left fifth intercostal space at the mid-clavicular line, V3 halfway between V2 and V4, V5 at the same level of V4 at the anterior axillary line, and V6 at the same level of V4 at the mid-axillary line; (*Figure 1*). The paECG were acquired by placing the precordial leads in the standard precordial lead position in the anterior thorax and with the patient laying in prone position.

### Computed tomography images

In order to measure the distance between the centre of the heart and the estimated ECG lead positions, 3D computed tomography (3D-CT) measurements in prone and supine positions were performed on eight of the volunteer subjects using Terarecon (Terarecon, Inc., Durham, NC, USA). Those healthy volunteers were randomly selected within the control group and were given written informed consent of the risks and adverse effects of radiation. The healthy volunteers were imaged without contrast administration or electrocardiographic gating in the following CT scanners (GE Optima 660, GE Lightspeed VCT, Siemens Somatom Sensation 16, and GE Optima CT 540). Images were reconstructed at 2.5–3.0 mm slice thickness.

**Figure 2:**
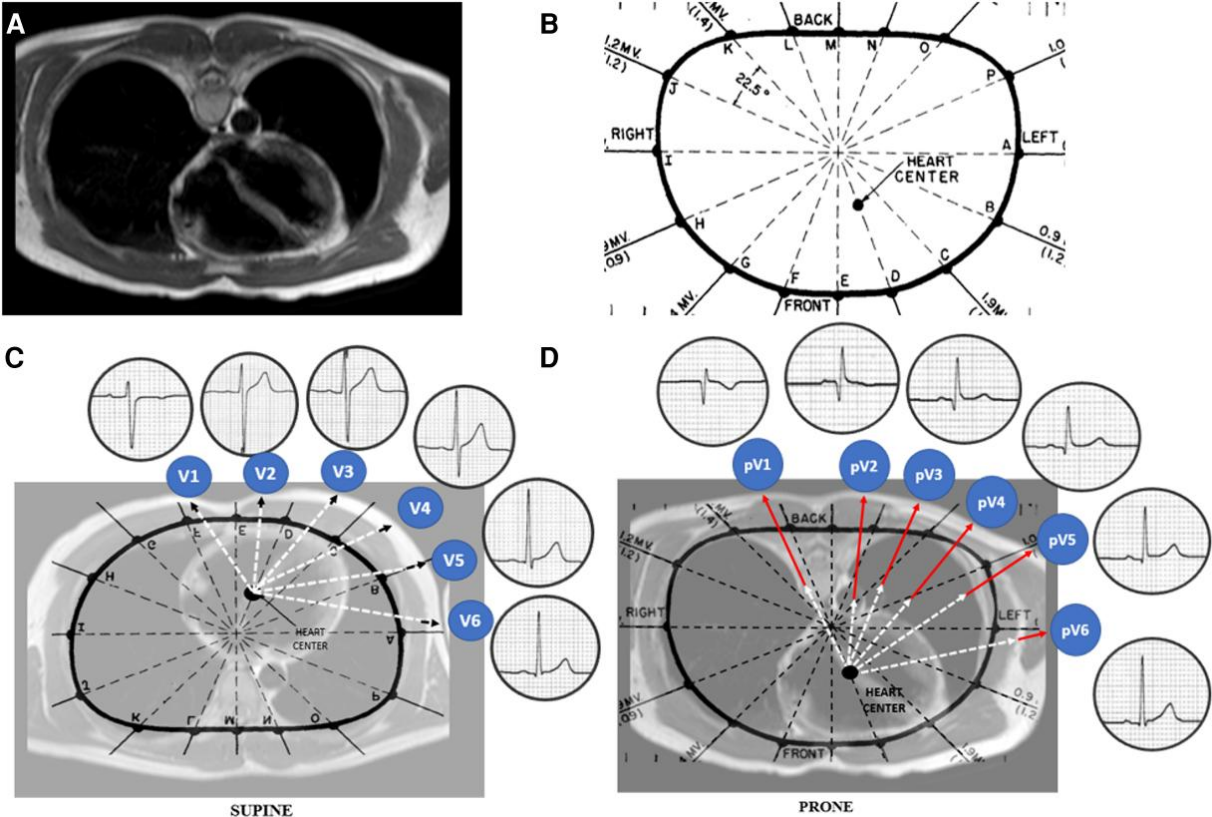
Assessment of the electroanatomic position of the heart. Difference in the distance from the centre of ventricular depolarization of the heart to the precordial electrodes in the supine and in prone position by chest magnetic resonance images. (*A*) Prone chest magnetic resonance imaging of a healthy volunteer. (*B*) Localization of the centre of ventricular depolarization (centre of the heart). (*C*) Measurement of the distance from the centre of the heart to the standard supine lead position (dotted arrows); see QRS morphology in supine position. (*D*) Measurement of the distance from centre of the heart to posterior prone lead position (dotted arrows represent the distance measured to standard lead position when supine; straight arrows show the difference in distance between anterior and posterior lead placement); see QRS morphology in prone back position. [Reproduced from ‘Frank E. Determination of the electrical centre of ventricular depolarization in the human heart. *Am Heart J* 1955;**49**:670–92’ with permission of the publisher].

First, we defined the electrical centre of ventricular depolarization of the heart following the analysis of Frank,^8^ who demonstrated that its magnitude, orientation and location can be calculated with an integration over the bounding surface of the resultant dipole of a system of sources and sinks inside a finite volume conductor. This mathematical and physical method, is used for locating the ‘heart vector’ or the resultant dipole of moment of the human heart was checked in 2D and 3D electrolytic tank models of human thorax, finding an accuracy of ±5 mm anatomically and ±0.2 mV electrically.^9^ We confirmed the location of the centre of the heart by superposing a standard figure with the calculated location by Frank et al, over the CT images of the eight subjects in the transverse plane (*Figure 2*). In all the patients, the estimated centre of the heart correlated with the proximal one-third and distal two-thirds of the interventricular septum in the axial plane after rotating the image to align with the interventricular septum axis. All measurements were performed by two independent CT readers with >5 years of experience (M.G., L.S.).

**Figure 3:**
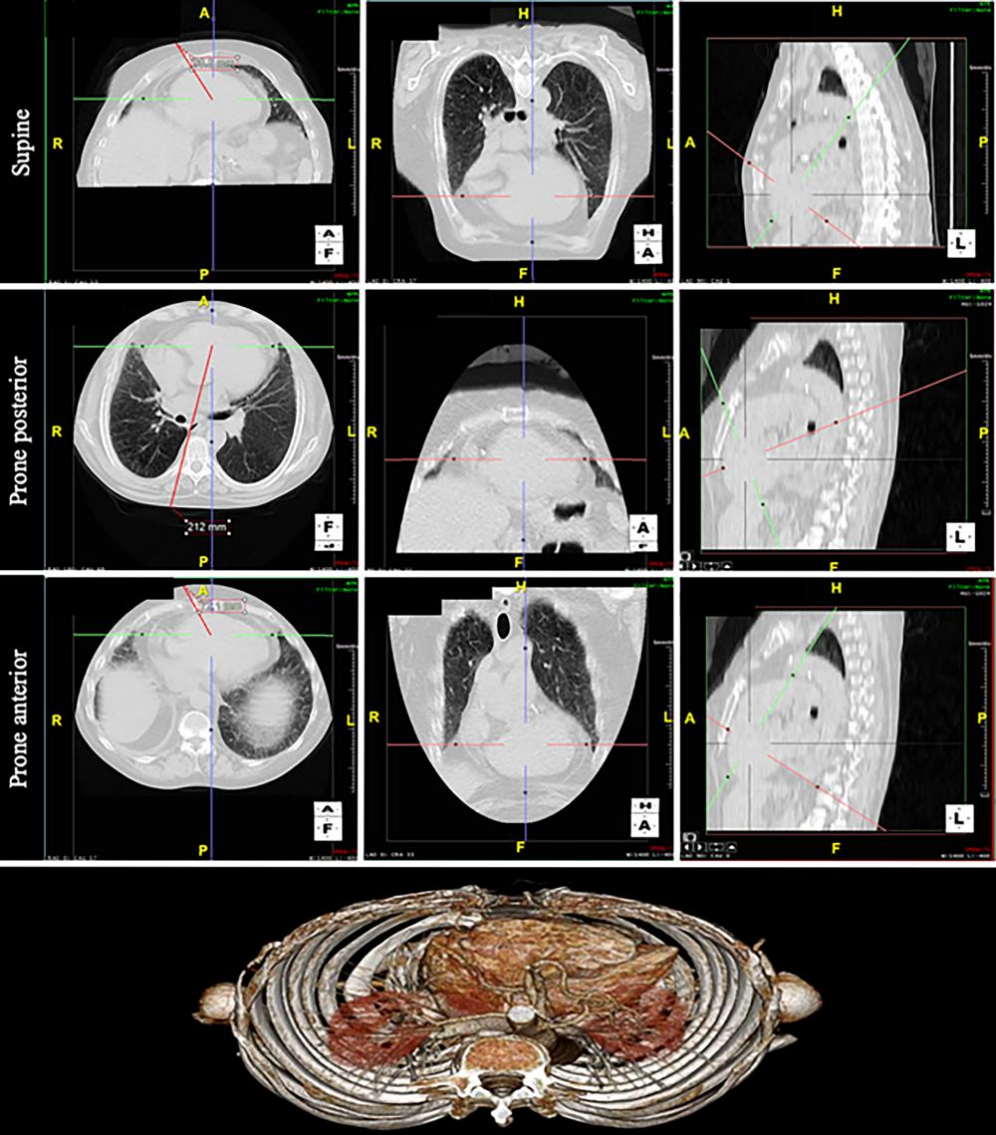
3D-computed tomography measurements: Axial (left), coronal (centre), sagittal (right), and 3-D volume rendered (bottom) chest images without contrast. Top panel showing the distance between the centre of the heart and the precordial leads in supine position. Central panel showing the distance between the centre of the heart and the precordial leads placed on patient’s back in prone position. Lower panel illustrating the distance between the centre of the heart and the precordial leads in prone anterior position (see Methods for details on measurements).

For measurement in prone back, we aligned the sagittal plane to the seventh spinal process and measured in the axial plane the distance to pbV1 (paraspinal right) and pbV2 (paraspinal left). Then the sagittal plane was aligned to the eighth spinal process and measured the distance to pbV4 (mid-scapular), pbV5 (posterior axillary line), and pbV6 (mid-axillary line) in the axial plane. Subsequently, we aligned the sagittal plane between the seventh and eighth spinal processes and measured, in the axial plane, the distance to pbV3 (between pbV2 and pbV4; *Figure 3*).

For the measurements in supine position, we aligned the sagittal plane to the fourth intercostal space and measured the distance to V1 (parasternal right) and V2 (parasternal left) in the axial view. We then aligned the sagittal plane to the fifth intercostal space and measured the distance to V4 (mid-clavicular), V5 (anterior axillary line), and V6 (mid-axillary line) in the axial plane. Subsequently, we aligned the sagittal plane between the fourth and fifth intercostal space and measured in the axial plane the distance to V3 (between V2 and V4; *Figure 3*).

In addition, the distances from the cardiac apex to the anterior chest (precordial leads) and to the posterior chest wall (posterior prone leads) were measured during prone and supine position.

### Data collection

Baseline characteristics were collected for all subjects. All ECGs performed in prone back, supine, and prone anterior positions were reviewed individually (J.R., J.C.D.). Values for heart rate (HR), PR duration, QRS duration, and QTc interval were obtained from the computer ECG reading. The QRS voltages (defined as the sum of all positive and negative deflections in the QRS) in all the precordial leads (V1–V6) were manually measured by the investigators for both anterior and posterior locations.^10^ The QRS axis was calculated as a vector using the QRS amplitudes in two limb leads.^11^ QRS and T-wave morphologies were evaluated individually by the investigators (J.R., J.C.D.). Statistical analysis can be seen in the supplementary information (see Supplementary material online, Supplementary information).

### Outcomes

The primary objective of this study was to compare ECG characteristics, including intervals, QRS morphology, QRS amplitude, ST segment, T-wave deviation, and QRS axis in the prone back and prone anterior position with those in the supine position. Secondary objectives included comparing the aforementioned parameters in the prone back position between patients with ARDS and healthy volunteers and differences in ECG characteristics between prone back and paECG acquired in healthy volunteers.

## Results

### Patient characteristics

Eighty-five individuals were included in the analysis (45 patients with ARDS and 40 healthy volunteers). A total of 210 ECGs were analysed: 85 pbECG, 85 sECG, and 40 paECG. From the entire cohort, 52.9% were males, the mean age was 48.8 ± 17 years, and the mean body mass index (BMI) was 27.9 ± 5.5 kg/m^2^; baseline characteristics are summarized in *Table 1*.

**Table 1:** Patient baseline characteristics

	ARDS group (*n* = 45)	Healthy volunteers (*n* = 40)	*P*-value (*t*-test)
Male sex, *n* (%)	26 (57.8)	19 (48.5)	*P* = 0.34
Age, year (mean ± SD)	61.8 ± 12.1	34.5 ± 7	***P* < 0.00001**
BMI (mean ± SD)	30.5 ± 6	24.9 ± 2.6	***P* < 0.00001**
*ECG characteristics: (supine position) mean*			
HR*	85.3 ± 16.8	68.7 ± 10.4	***P* < 0.00001**
ȃFemale	80.7 ± 13.6	69.2 ± 10.0	***P* < 0.00001**
ȃMale	88.8 ± 18.4	68.2 ± 11.2	***P* < 0.00001**
PR (ms)*	157.7 ± 20.9	151.2 ± 22	*P* = 0.2169
ȃFemale	163.6 ± 21.8	152.8 ± 24.4	*P* = 0.1460
ȃMale	153.1 ± 18.6	150.9 ± 19.6	*P* = 0.7107
QRS (ms)*	92.7 ± 19.2	91.7 ± 8.5	*P* = 0.7507
ȃFemale	88.9 ± 14.5	90.2 ± 8.4	*P* = 0.7460
ȃMale	95.5 ± 8.6	93.4 ± 8.6	*P* = 0.660
QTc (ms)*	451.5 ± 33.2	421.3 ± 19.7	***P* < 0.00001**
ȃFemale	460.6 ± 30.0	430.4 ± 15.1	***P* = 0.00069**
ȃMale	444.8 ± 33.8	411.2 ± 19.3	*P* = 0.00132
*Supine ECG*			
Lead V1 amplitude (mV)	1.08 ± 0.51	1.08 ± 0.35	*P* = 0.49
Lead V2 amplitude (mV)	1.38 ± 0.71	1.86 ± 0.66	***P* = 0.0006**
Lead V3 amplitude (mV)	1.60 ± 0.67	1.64 ± 0.65	*P* = 0.37
Lead V4 amplitude (mV)	1.65 ± 0.74	1.65 ± 0.48	*P* = 0.49
Lead V5 amplitude (mV)	1.42 ± 0.50	1.41 ± 0.43	*P* = 0.44
Lead V6 amplitude (mV)	1.44 ± 0.47	1.05 ± 0.31	*P* = 0.12
*Prone back ECG*			
Lead pV1 amplitude (mV)	0.35 ± 0.13	0.42 ± 0.12	*P* = 0.006
Lead pV2 amplitude (mV)	0.35 ± 0.15	0.38 ± 0.14	*P* = 0.08
Lead pV3 amplitude (mV)	0.46 ± 0.21	0.50 ± 0.17	*P* = 0.24
Lead pV4 amplitude (mV)	0.61 ± 0.26	0.65 ± 0.24	*P* = 0.45
Lead pV5 amplitude (mV)	1.04 ± 0.84	0.85 ± 0.27	*P* = 0.14
Lead pV6 amplitude (mV)	1.23 ± 0.48	1.11 ± 0.29	*P* = 0.20

Data for baseline characteristics and ECG characteristics of the ARDS group and the healthy volunteers group. Table shows the difference between the ARDS and the healthy volunteers. Text in bold identifies significant parameters. As multiple comparisons were performed, a Bonferroni test was used to get a correction of the type I error; a *p* ≤ 0.0008 was considered to be statistically significant.

ARDS, acute respiratory distress syndrome; BMI, body mass index; ECG, electrocardiogram; HR, heart rate; pV, prone back V; SD, standard deviation.

### Quantitative parameters

#### Electrocardiogram intervals and frontal plane axis

No significant differences were noted between pbECG and sECG in HR (79 ± 15.8 vs. 77.5 ± 16.4 bpm, *P* = 0.080), PR interval (156.6 ± 22.7 vs. 154.9 ± 21.5 ms, *P* = 0.948), QTc intervals (427.9 ± 29 vs. 437 ± 31.4 ms, *P* = 0.006), and QRS axis (21.8 ± 49.2 vs. 30.2 ± 47.0, *P* = 0.007). Conversely, although there were significant statistical differences in pbECG when compared with sECG in QRS duration (85.8 ± 17.5 vs. 92.2 ± 15 ms, *P* < 0.0001), the difference was not clinically relevant (*Table 2*).

**Table 2:** Univariate analysis of electrocardiogram findings on acute respiratory distress syndrome and healthy volunteers (*n* = 85)

ECG characteristic (mean ± SD unless specified)	Prone back	Supine	*P*-value (*t*-test)
Heart rate	79 ± 15.8	77.5 ± 16.4	*P* = 0.08
PR interval (ms)	156.6 ± 22.7	154.9 ± 21.5	*P* = 0.9481
QRS duration (ms)	85.8 ± 17.5	92.2 ± 15	***P* < 0.0001**
QTc (Bazett)	427.9 ± 29	437.3 ± 31.4	*P* = 0.006
Lead V1 amplitude (mV)	0.38 ± 0.13	1.08 ± 0.44	***P* < 0.0001**
Lead V2 amplitude (mV)	0.35 ± 0.15	1.61 ± 0.73	***P* < 0.0001**
Lead V3 amplitude (mV)	0.48 ± 0.19	1.62 ± 0.66	***P* < 0.0001**
Lead V4 amplitude (mV)	0.63 ± 0.24	1.65 ± 0.63	***P* < 0.0001**
Lead V5 amplitude (mV)	0.95 ± 0.64	1.41 ± 0.47	***P* < 0.0001**
Lead V6 amplitude (mV)	1.17 ± 0.40	1.10 ± 0.41	*P* = 0.054
QRS axis (deg)	21.8 ± 49.2	30.2 ± 47.0	*P* = 0.007

Text in bold identifies significant parameters. As multiple comparisons were performed, a Bonferroni test was used to get a correction of the type I error; a *p* ≤ 0.0008 was considered to be statistically significant.

deg, degree; ECG, electrocardiogram; SD, standard deviation.

#### QRS amplitude and morphology

**Figure 4:**
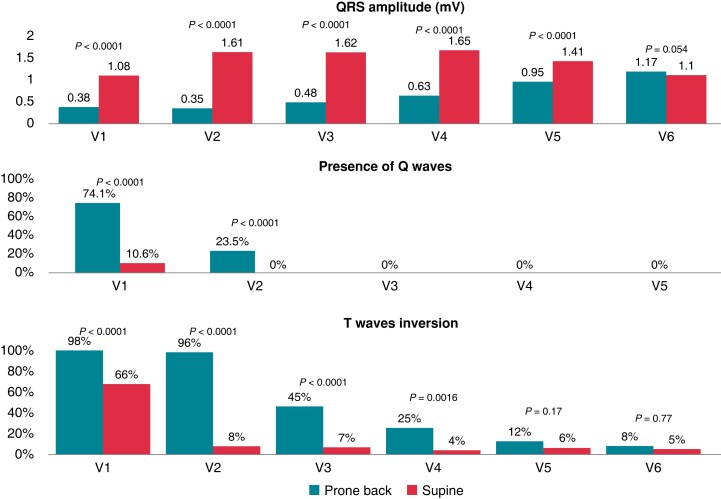
Prone back vs. supine position. Differences in QRS amplitude, Q waves, and T-wave abnormalities between prone and supine position. As multiple comparisons were performed, a Bonferroni test was used to get a correction of the type I error; a *p* ≤ 0.0008 was considered to be statistically significant.

In the overall cohort, there were significant differences in QRS amplitudes between prone back and supine positions, with lower voltages observed in the pbECG Leads V1–V5 [V1: 0.38 vs. 1.08 mV (absolute reduction: 59.5%), *P* < 0.0001; V2: 0.35 vs. 1.61 mV (absolute reduction: 75%), *P* < 0.0001; V3: 0.48 vs. 1.62 mV (absolute reduction: 64.1%), *P* < 0.0001; V4: 0.63 vs. 1.65 mV (absolute reduction: 55.4%), *P* < 0.0001 and V5: 0.95 vs. 1.41 mV (absolute reduction: 25.1%), *P* < 0.0001, respectively]. There was no significant difference in Lead V6 (*P* = 0.054; *Figure 4*, *Table 2*, and Supplementary material online, *Figure S1* and *Table S1*). No statistical difference was noted in the QRS amplitude between limb leads (I, II, III, aVL, aVR, and aVF) in pbECG and sECG.

### Qualitative parameters

#### Presence of Q waves

In the total cohort, an important finding was the presence of new prominent Q waves in the prone back leads. Q waves were found in 74.1% of the study population in lead pbV1 compared with 10.6% in the lead sV1 (*P* < 0.0001). In Lead pbV2, 23.5% of the study population had Q waves vs. 0% in Lead sV2 (*P* < 0.0001).

Non-pathological Q waves were seen in the other prone back leads. For Lead pbV3, 78.8% of the study populations had Q waves vs. 3.5% in Lead sV3 (*P* < 0.0001). Additionally, 70.6% had Q waves in pbV4 vs. 7.1% in lead sV4 (*P* < 0.0001). These ‘normal’ Q waves had a duration <40 ms in all cases, with the highest amplitude in Lead V1 which progressively declined towards Lead pbV4 (0.22 ± 0.1 mV in Lead pbV1; 0.1 ± 0.07 mV in Lead pbV2; 0.06 ± 0.03 mV in Lead pbV3; 0.04 ± 0.02 mV in Lead pbV4). No significant differences were observed in Leads V5 and V6 between the prone and supine position (*Figure 4*, see Supplementary material online, *Figure S1* and *Tables S2A and B*).

#### T-wave morphology

Significant differences in T-wave morphology in the total cohort were noted in the prone vs. supine position, with T-wave abnormalities (T-wave inversion or flat T waves) observed in Leads V1 (98 vs. 66%; *P* < 0.0001), V2 (96 vs. 8%; *P* < 0.0001), and V3 (45 vs. 7%; *P* < 0.0001). No significant differences in T-wave morphology in V4, V5, and V6 between prone back and supine ECGs (*P* = 0.0016, *P* = 0.17, and *P* = 0.77, respectively) were observed. Moreover, no differences were found between males and females (*Figure 4*, see Supplementary material online, *Figure S1* and *Tables S2A and B*).

### Subanalysis

#### Healthy volunteers vs. patients with acute respiratory distress syndrome

A subanalysis was performed to establish whether there were significant differences in the measured variables between healthy volunteers and patients with ARDS. Although no difference was observed in PR interval and QRS duration, there were significant differences in HR and QTc intervals between both groups (*Table 1*, see Supplementary material online, *Table S5*).

The QRS voltage reductions in prone back position, compared with the supine position, were observed in Leads pbV1–pbV5, both in the healthy volunteers and in the ARDS cohort (*Tables 1* and *3*, see Supplementary material online, *Tables S1A–C and 5*).

**Table 3:** Univariate analysis of electrocardiogram findings on supine, prone with anterior precordial lead, and prone with posterior precordial lead electrocardiograms for healthy volunteers (*n* = 40)

ECG characteristic (mean ± SD unless specified)	Supine	Prone (anterior leads)	Prone (posterior leads)	*P*-value (*t*-test, supine vs. prone back)	*P*-value (*t*-test, supine vs. prone anterior)
Lead V1 amplitude (mV)	1.08 ± 0.36	1.00 ± 0.38	0.42 ± 0.12	***P* < 0.0001**	*P* = 0.330
Lead V2 amplitude (mV)	1.88 ± 0.67	1.49 ± 0.64	0.38 ± 0.13	***P* < 0.0001**	*P* = 0.010
Lead V3 amplitude (mV)	1.64 ± 0.65	1.70 ± 0.58	0.50 ± 0.17	***P* < 0.0001**	*P* = 0.701
Lead V4 amplitude (mV)	1.65 ± 0.48	1.66 ± 0.42	0.65 ± 0.22	***P* < 0.0001**	*P* = 0.960
Lead V5 amplitude (mV)	1.41 ± 0.43	1.72 ± 1.55	0.85 ± 0.27	***P* < 0.0001**	*P* = 0.229
Lead V6 amplitude (mV)	1.04 ± 0.31	1.10 ± 0.31	1.12 ± 0.29	*P* = 0.039	*P* = 0.464
QRS axis (deg)	55.9 ± 29.9	42.7 ± 39.8	45.2 ± 37.9	***P* = 0.0001**	*P* = 0.097

Data from the healthy volunteer group showing the difference between the supine, prone back, and prone anterior ECG parameters. Text in bold identifies significant parameters. As multiple comparisons were performed, a Bonferroni test was used to get a correction of the type I error; a *p* ≤ 0.0008 was considered to be statistically significant.

deg, degree; ECG, electrocardiogram; SD, standard deviation.

#### Supine vs. prone anterior lead position

The ECGs were acquired in the prone anterior position in the group of 40 healthy volunteers as an alternative to prone back lead positioning. No difference in the QRS voltage was seen when comparing prone anterior and supine position (see *Table 3*). Importantly, when placed in the prone anterior position, Q waves were not observed in precordial leads as opposed to pbECG (see Supplementary material online, *Table S2A*).

#### Computed tomography images

A subanalysis of chest CT images in eight healthy volunteers was performed. There were significant differences between the distance from the defined centre of ventricular depolarization of the heart to the estimated precordial electrodes in the prone and supine position (V1: 196.3 vs. 95.6 mm, *P* < 0.0001; V2: 186.38 vs. 77.4 mm, *P* < 0.0001; V3: 183.25 vs. 88.2 mm, *P* < 0.0001; V4: 181.5 vs. 95.3 mm, *P* < 0.0001; V5: 179 vs. 111 mm, *P* < 0.0001; V6: 160 vs. 132 mm, *P* < 0.0001, respectively; *Figure 2*).

**Figure 5:**
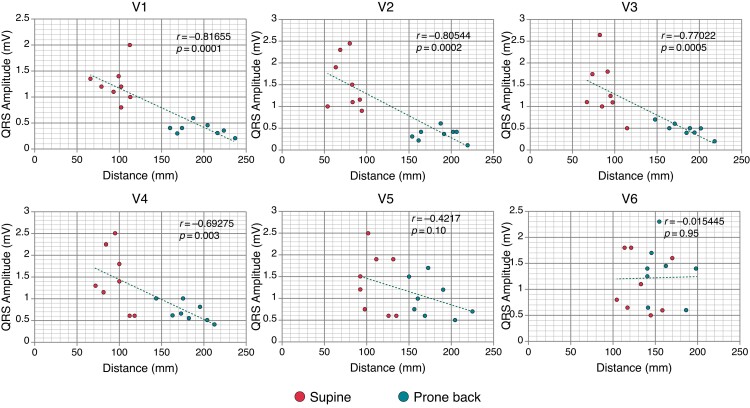
Correlation between QRS amplitude and distance to electrodes. Scatter plots delineating the V1–V6 values and their relationship between QRS amplitude and lead position distance by Pearson’s correlation analysis. *n* = 8. As multiple comparisons were performed, a Bonferroni test was used to get a correction of the type I error; a *p* ≤ 0.0008 was considered to be statistically significant.

To determine if these differences could explain the changes in QRS amplitude observed between both supine and prone positions, a Pearson’s correlation analysis was performed. This showed a significant inverse correlation in Leads V1–V3 but not for Leads V4–V6 (V1: *r* = −0.816, *P* = 0.0001; V2: *r* = −0.805, *P* = 0.0002; V3: *r* = −0.77, *P* = 0.0005; V4: *r* = −0.692, *P* = 0.003; V5: *r* = −0.0422, *P = 0*.10; V6: *r* = −0.02, *P* = 0.95; *Figure 5*).

In addition, the distances from the cardiac apex to the anterior chest (precordial leads) were measured in the prone and supine position, and there were no significant differences between the measurements (see Supplementary material online, *Table S4*).

#### Interobserver and intraobserver variabilities

The interobserver agreement was excellent for the measurements made by the authors (QRS amplitude, T-wave morphologies, and CT measurements). The intraobserver agreement was also excellent (Supplementary information).

## Discussion

Prone positioning has been proved to reduce mortality in ARDS,^1^ and this includes beneficial outcomes in patients with COVID-19.^12^ Additionally, prone positioning is used in surgeries, including spinal and orthopaedic surgeries and those involving the retroperitoneum. However, prone positioning comes with certain challenges in patient care and monitoring. Given the high risk of cardiovascular events in patients undergoing invasive procedures or with mechanical ventilation for ARDS, the correct interpretation of the 12-lead ECG in the prone position is crucial for proper patient care. Thus, our study has several important findings which should be taken into consideration and that could allow for standardization of ECG interpretation in the prone position:

1. QRS amplitudes in most of posterior leads (pbV1–pbV5) are significantly reduced when compared with precordial leads.
2. New pseudo-Q waves are frequently observed from pbV1–pbV2, while T wave inversion is present in most patients in lead pbV1-pbV2, almost half of the patients in pbV3 and in only 25% of patients extending to lead pbV4. Changes in other derivations should prompt further evaluation.
3. 3D-CT reconstruction measurements showed a significant inverse correlation between QRS amplitude and the distance from the centre of the heart to the estimated lead positions.
4. Placing the leads on the anterior thorax during prone position is not associated with changes in QRS morphology, voltage, or T-wave polarity compared with the supine position.
5. Prone position is associated with statistical differences in QRS duration compared with supine position. However, these differences are not clinically significant.

### Electrocardiogram changes in the prone position

We demonstrated that prone back lead placement resulted in significant reductions in QRS amplitude in leads pbV1–pbV5 compared with the supine position (*Figures 1* and *4*, see Supplementary material online, *Figure S1*). Although other studies have already suggested that body position can have significant effects on QRS morphology and amplitude^13^ and it is widely accepted that ECG wave amplitude is greatly determined by the distance between the electrode and the electrical centre of the recorded chamber, by using 3D-CT images with precise measurements, we are the first to corroborate and objectively demonstrate this phenomenon (inverse correlation between the distance from the centre of ventricular depolarization of the heart to the electrodes’ expected location and the QRS amplitude; *Figure 5*). Also, we have demonstrated that these changes in pbECG can be explained by the increased impedance (due to the interposing lung tissue) and the ventral and caudal shift observed in the heart during prone position^14^

In our study, QRS amplitudes in pbV1–pbV5 leads were <1.0 mV and the mean QRS amplitude of pbV1–pbV6 was 0.66 mV (*Figure 4*, *Tables 1–3*, see Supplementary material online, *Table S5*). Considering that ‘low voltage’ on a standard 12-lead ECG is defined as a peak-to-peak QRS amplitude <0.5 mV in limb leads and/or <1.0 mV in the precordial leads,^15^ the QRS amplitudes of posterior leads in individuals undergoing ECGs in prone position would essentially meet the criteria for low voltage. Consequently, low voltage in the prone back leads should be expected and not mistaken with conditions that present with low voltage QRS, such as pericardial effusions, infiltrative disease, COPD, etc.

More importantly, Q waves and inverted or flat T waves were frequently observed in the prone back lead configuration (pbV1–pbV2 and pbV1–pbV3 leads, respectively; *Figures 1* and *4*). As a result, these changes should be expected and not misinterpreted with findings associated with myocardial ischaemia. These variations are explained by the resultant modification in QRS vector with the change in cardiac position and posterior electrode placement, whereby ventricular depolarization is directed away from Leads pbV1 to pbV3, resulting in the apparition of Q waves and low amplitude R waves in these leads in the prone back position as opposed to the supine position^16^ Thus, when using prone back leads, Q waves should be considered normal in Leads V1–V2 and inverted or flat T waves should be considered normal in Leads V1–V3, but their presence in other posterior leads should be considered abnormal and should prompt further evaluation, especially in patients in whom myocardial ischaemia is suspected. Therefore, we strongly recommend considering these findings to avoid unnecessary cardiac imaging testing and/or invasive cardiovascular procedures in patients with ECGs obtained in a prone position.

There were significant differences in QRS and QTc between prone back and sECG. While the exact mechanism underlying this finding could not be elucidated, two possible explanations may exist: first, the change in heart morphology (which assumes a more globular position in which a larger area is in contact with the thoracic wall),^14^ along with a change in autonomic control of the heart, could explain the changes both in QRS duration and in QTc during prone position. Indeed, prone position has been associated with changes in autonomic modulation both in infants and in adults,^17,18^ and as such could have a significant impact on repolarization.

Moreover, patients with ARDS were found to have significantly longer QTc intervals when compared with healthy volunteers. This could simply be explained by either difference in individual patient characteristics and clinical condition, or prolongation of the QT induced by medications, electrolytes disturbances or even coronavirus infection.^19^

Previously published studies of COVID-19 patients, as the one by Nguyen *et al*.^16^ described a reduction in mean QRS amplitudes in the posterior leads (mainly pbV1–pbV5), reduction of R-wave amplitude in Leads pbV1–pbV4, and presence of Q waves in Leads pbV1–pbV3 in pbECG compared with the supine position. Similarly, Chieng *et al*.,^20^ published their experience of pbECG in patients with basal ECG changes, finding similar QT prolongation, QRS morphology, and T-wave changes as reported in our study.

However, our study offers a greater degree of certainty and reliability because it is the first to explain the aforementioned changes in the ECG during prone position through measurements made with 3D-CT. Through these measurements, we managed to confirm the location of the centre of the heart proposed by Frank,^8^ and aligned it in the sagittal plane in order to determine its distance to each electrodes’ expected location (*Figure 2*). Similarly, an individual measurement was made for each of the leads without making arbitrary grouping. Finally, QRS voltage measurements were made following the international standards by measuring from the nadir of the QRS complex to its peak.^15^

### Limitations

Our study has some limitations which should be taken into consideration before interpreting our results. Although the small sample size may limit the generalizability of our findings and could create discrepancies in some results, it is important to understand that each patient serves as their own control, and that the objective was to determine the difference of each patient between the different positions. There were differences in the baseline characteristics between both patient groups (age and BMI), which may have accounted for some of the differences in QRS amplitude, as well as QRS and T-wave morphology. As this study included patients from the USA and Colombia, it is important to specify that patients could differ in some characteristics due to demographic variations. However, taking into consideration that Latinos are currently the largest ethnic minority in the USA, and that they are frequently underrepresented in clinical trials, inclusion of a Latino population may increase the generalizability of our results.^21^ Prone anterior ECGs were not measured in the ARDS groups in order to reduce the manipulation and discomfort of those patients. Moreover, the utility of prone ECG in the identification of specific rhythm disorders or conduction disturbances is yet to be elucidated.

## Conclusions

Low QRS amplitude, or Q waves and T wave abnormalities observed in prone position ECG should not be misinterpreted as low voltage conditions (effusions, infiltrative disease) or anteroseptal myocardial injury, respectively. These changes can be explained by an increased impedance (due to the interposing lung tissue), and by the increased distance between the electrodes to the centre of the heart. Considering no significant changes were seen in prone anterior ECGs, it can be used as an alternative to supine and pbECG when possible.

### Clinical implications

- This study enhanced a more accurate interpretation of the prone back ECG.
- QRS amplitude in prone back ECG should not be mistakenly confused with conditions that present with low voltage QRS, such as pericardial effusions, infiltrative disease, and COPD.
- Inverted or flat T waves in prone back ECG should be expected in V1–V3 and not misinterpreted with findings associated with myocardial ischaemia.
- Q waves should be considered normal in Leads pbV1–pbV2 when using prone back leads, but their presence in V3–V6 should be considered abnormal and should prompt further evaluation.

## Supplementary material

Supplementary material is available at *Europace* online.

## Supplementary Material

euac099_Supplementary_Data

## Data Availability

The data underlying this article are available in the article and in its online Supplementary material.
