# Supplementary material for: Anatomical considerations and clinical interpretation of the 12-lead ECG in the prone position: a prospective multicentre study
Source: Europace. 2022 Oct 5;25(1):175–84. doi: 10.1093/europace/euac099 (PMC10103558; doi:10.1093/europace/euac099)
Supplement: euac099_Supplementary_Data [file euac099_supplementary_data.zip › Prone Supplementary Files clean version JR.docx]

**Supplementary material**

**Supplementary information**

**Statistical Analysis**

Statistical analysis was performed using SAS version 9.4 (SAS Institute Inc., Cary, NC, USA). The first authors and the corresponding author had full access to all the studied data and take responsibility for its integrity and the data analysis. Univariate analysis of ECG data was performed. Interval and axis measurements and lead voltages were compared between supine and prone ECGs for both groups. Measurements were compared using Student’s t-test, Pearson’s correlation coefficient and chi^2^ analysis. As multiple comparisons were performed, a Bonferroni test was used to get a correction of the type I error; a p ≤ 0.0008 was considered to be statistically significant. For ECG measurements and CT measurements, interobserver and intraobserver variability was analyzed using Shrout-Fleiss intraclass correlation.

**Interobserver and Intraobserver Variability**

The interobserver agreement was excellent for the measurements made by the authors (QRS amplitude, T wave morphologies and CT measurements) [the Shrout-Fleiss interobserver correlation coefficient for the whole set of measurements: 0.961, interobserver correlation coefficient for prone back: 0.895, interobserver correlation coefficient for supine: 0.900, interobserver correlation coefficient for prone anterior: 0.903]. The intraobserver agreement was also excellent with a Shrout-Fleiss intraclass correlation coefficient of 0.967.


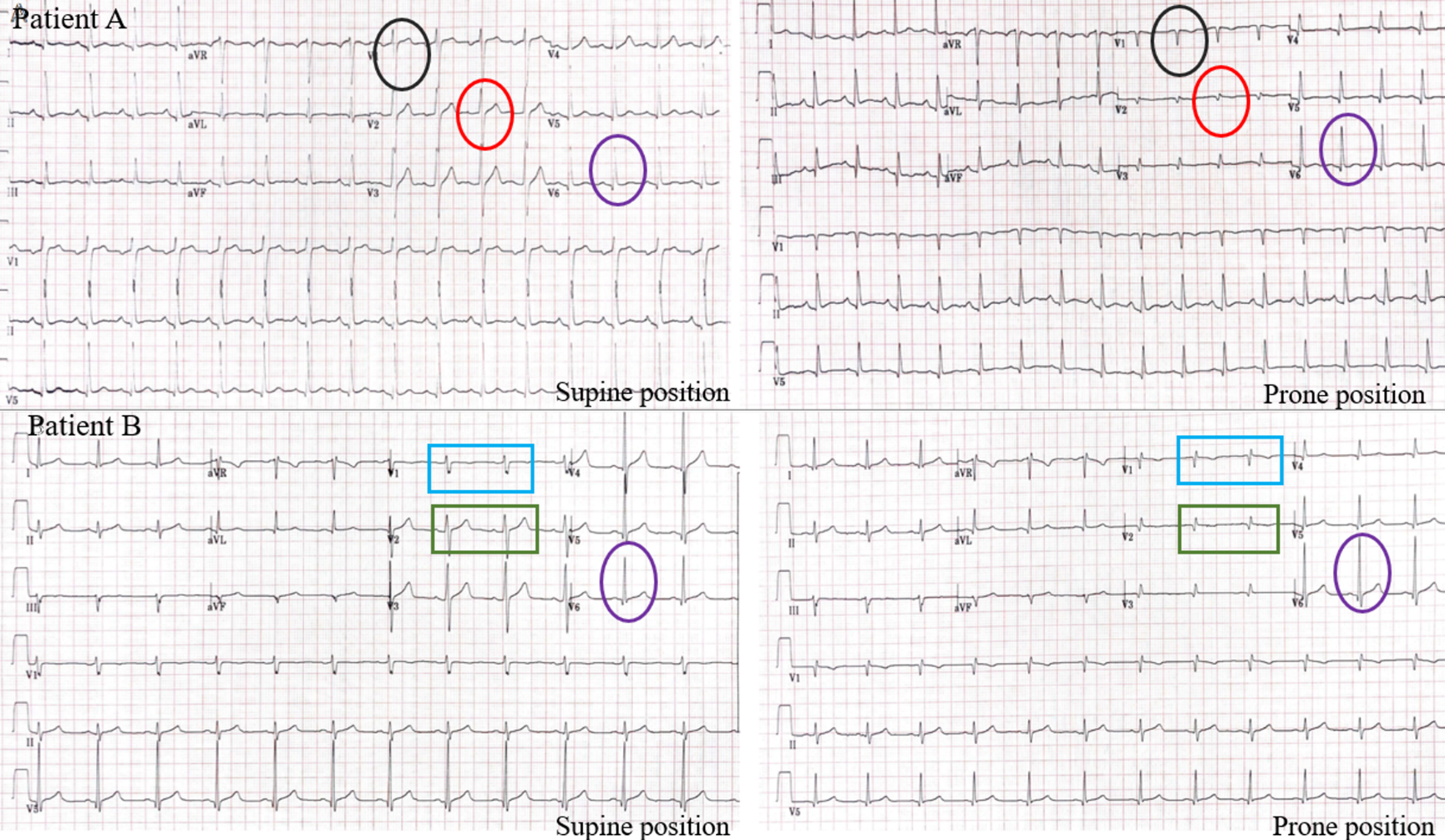
**Supplementary Figure 1.- Electrocardiogram of two representative patients** (A and B) in the supine position (on the left side) and prone position (on the right side). Notice the difference in QRS amplitude (red circle), QRS morphology (blue square), T waves abnormalities (green square) and presence of Q wave (black circle) in supine vs. prone position. Also notice the similarities between QRS morphology and amplitude in lead V6 between supine and prone position.

**Supplementary Table 1a.-** QRS voltage change between supine and prone back lead placement in all patients

| Patient | V1 | V2 | V3 | V4 | V5 | V6 |
| --- | --- | --- | --- | --- | --- | --- |
| 1 | -50.0% | -66.7% | -68.4% | -72.0% | -60.9% | 3.4% |
| 2 | -70.6% | -87.1% | -72.2% | -72.0% | -25.0% | -18.5% |
| 3 | -57.1% | -75.5% | -84.9% | -77.8% | -63.2% | 27.3% |
| 4 | -68.6% | -87.8% | -38.1% | -35.3% | -28.6% | 10.0% |
| 5 | -43.8% | -83.9% | -84.3% | -86.0% | -66.7% | -5.6% |
| 6 | -84.2% | -65.2% | -71.4% | -65.6% | -41.7% | 0.0% |
| 7 | -11.1% | -71.4% | -69.6% | -68.2% | -21.1% | -3.7% |
| 8 | -70.8% | -82.6% | -71.4% | -52.2% | -20.0% | 0.0% |
| 9 | -52.9% | -86.8% | -52.2% | -55.6% | -36.7% | 13.6% |
| 10 | -64.7% | -79.4% | -69.2% | -64.6% | -45.0% | -6.9% |
| 11 | -50.0% | -83.3% | -83.3% | -81.5% | -60.5% | -20.0% |
| 12 | -60.0% | -90.0% | -37.5% | -63.5% | -47.6% | 0.0% |
| 13 | -54.5% | -76.7% | -62.2% | -53.8% | -42.9% | -7.4% |
| 14 | -69.7% | -73.9% | -33.3% | -14.8% | -10.0% | 28.0% |
| 15 | -55.0% | -69.6% | -68.0% | -53.6% | -37.5% | 56.3% |
| 16 | -52.2% | -77.1% | -69.2% | -69.0% | -48.0% | 12.5% |
| 17 | -70.0% | -78.3% | -79.5% | -60.7% | 27.3% | -23.8% |
| 18 | -77.8% | -70.0% | -60.0% | -56.5% | -45.5% | 42.9% |
| 19 | -50.0% | -87.1% | -89.7% | -80.0% | -51.7% | 33.3% |
| 20 | -76.5% | -87.5% | -84.9% | -50.0% | -16.7% | 20.0% |
| 21 | -50.0% | -68.8% | -46.7% | -35.7% | -16.0% | 42.1% |
| 22 | -61.1% | -83.3% | -82.5% | -58.3% | -23.8% | 46.7% |
| 23 | -50.0% | -70.0% | -68.2% | -70.4% | -40.0% | 14.3% |
| 24 | -70.0% | -76.9% | -63.0% | -63.0% | -54.2% | -5.9% |
| 25 | -70.4% | -84.2% | -30.0% | -44.4% | -21.1% | 17.2% |
| 26 | -68.2% | -89.5% | -55.6% | -22.7% | -16.7% | -36.1% |
| 27 | -76.0% | -81.3% | -35.3% | -52.9% | -62.7% | -3.0% |
| 28 | -65.5% | -81.1% | -82.4% | -73.3% | -58.3% | 16.7% |
| 29 | -69.2% | -88.4% | -53.8% | -51.4% | -24.2% | 11.5% |
| 30 | -43.8% | -77.5% | -81.5% | -76.1% | -40.7% | -5.6% |
| 31 | -56.5% | -89.7% | -78.9% | -73.7% | -51.5% | 8.7% |
| 32 | 11.1% | -84.4% | -73.7% | -53.3% | -40.7% | 10.5% |
| 33 | -55.0% | -61.5% | -36.4% | -13.6% | -20.0% | 11.8% |
| 34 | -12.5% | -73.9% | -70.0% | -65.2% | -42.9% | 58.3% |
| 35 | -70.0% | -80.0% | -85.3% | -53.3% | -21.7% | -6.3% |
| 36 | -52.4% | -67.5% | -75.0% | -69.7% | -52.0% | 0.0% |
| 37 | -61.9% | -75.0% | -45.5% | -51.7% | -35.7% | 14.3% |
| 38 | -71.4% | -81.8% | -93.5% | -78.6% | -64.3% | 0.0% |
| 39 | -65.0% | -79.3% | -14.3% | -40.7% | -25.9% | 13.6% |
| 40 | -50.0% | -36.4% | -37.3% | -20.0% | -37.5% | 23.5% |
| 41 | -73.7% | -90.0% | -83.3% | -65.0% | -41.7% | 15.4% |
| 42 | -62.5% | -84.2% | -80.0% | -76.9% | -33.3% | -25.0% |
| 43 | -73.3% | -50.0% | -27.3% | -27.3% | -25.0% | 112.5% |
| 44 | -71.4% | -83.3% | -91.7% | -91.7% | -61.9% | 62.5% |
| 45 | 0.0% | -75.0% | -37.5% | -18.2% | -52.2% | 18.8% |
| 46 | -42.9% | -50.0% | -36.4% | -36.4% | -50.0% | 0.0% |
| 47 | -35.7% | -74.2% | -72.4% | -73.1% | 100.0% | -21.4% |
| 48 | -76.5% | -81.8% | -50.0% | -15.4% | -43.5% | 27.3% |
| 49 | -80.0% | -62.5% | -68.8% | -68.2% | -65.0% | -28.6% |
| 50 | -55.6% | -80.0% | -81.3% | -60.0% | -53.3% | 0.0% |
| 51 | -84.6% | -90.9% | -85.2% | -87.5% | -50.0% | -53.8% |
| 52 | -62.5% | -83.3% | -88.9% | -75.0% | -56.3% | 45.5% |
| 53 | -76.9% | -71.4% | -66.7% | -70.0% | -64.3% | -5.9% |
| 54 | -81.3% | -50.0% | -35.7% | -15.4% | -22.7% | 7.7% |
| 55 | -77.8% | -71.4% | 16.7% | 25.0% | -38.5% | 171.4% |
| 56 | -75.0% | -80.0% | -54.5% | -53.8% | -16.7% | -22.2% |
| 57 | -84.6% | -75.0% | -33.3% | 42.9% | -64.7% | 20.0% |
| 58 | -70.0% | -40.0% | -28.6% | -40.0% | 0.0% | -42.9% |
| 59 | -62.5% | -66.7% | -66.7% | -70.0% | -55.0% | 0.0% |
| 60 | -44.4% | -66.7% | -78.6% | -61.5% | -33.3% | -17.6% |
| 61 | -55.6% | -89.3% | -87.0% | -81.5% | -44.4% | -54.2% |
| 62 | -14.3% | -71.4% | -82.6% | -81.8% | -38.5% | 29.4% |
| 63 | -62.5% | -69.2% | -76.5% | -64.3% | 0.0% | -11.1% |
| 64 | -85.0% | -55.6% | 0.0% | 33.3% | -53.8% | 166.7% |
| 65 | -75.0% | -88.0% | -83.3% | -80.0% | -9.1% | -15.8% |
| 66 | -62.5% | -71.4% | -80.0% | -57.1% | -52.9% | 0.0% |
| 67 | -81.8% | -81.8% | -80.0% | -77.8% | -32.0% | 71.4% |
| 68 | -75.0% | -57.1% | -44.4% | -44.4% | -7.1% | -44.4% |
| 69 | 0.0% | -57.1% | -81.0% | -73.7% | -41.7% | -11.1% |
| 70 | -76.9% | -88.0% | -81.8% | -76.2% | -33.3% | -33.3% |
| 71 | -55.6% | -65.0% | -71.0% | -42.9% | -25.0% | 27.8% |
| 72 | 0.0% | -80.0% | -77.8% | -62.5% | -61.9% | 22.2% |
| 73 | -81.8% | -93.3% | -81.8% | -33.3% | -52.2% | 20.0% |
| 74 | -40.0% | -72.7% | -87.0% | -88.9% | -50.0% | 100.0% |
| 75 | -75.0% | -75.0% | -69.2% | -38.5% | 100.0% | 21.4% |
| 76 | -71.4% | -85.7% | -87.0% | -80.0% | -43.5% | -7.7% |
| 77 | -54.5% | -82.6% | -70.6% | -68.2% | -65.0% | 8.3% |
| 78 | -62.5% | -50.0% | -91.7% | -86.7% | -53.3% | 28.6% |
| 79 | -60.0% | -83.3% | -88.9% | -75.0% | -50.0% | 75.0% |
| 80 | -75.0% | -84.2% | -76.5% | -54.5% | -56.3% | 40.0% |
| 81 | -57.1% | -83.3% | -80.0% | -73.3% | -64.3% | -10.0% |
| 82 | -62.5% | -60.0% | -40.0% | -18.2% | -22.7% | 0.0% |
| 83 | -50.0% | -66.7% | -73.3% | -70.6% | -38.5% | -8.3% |
| 84 | -50.0% | -63.6% | -66.7% | -60.0% | -16.7% | 27.8% |
| 85 | -84.6% | -61.5% | 14.3% | -16.7% | -64.7% | 8.3% |

**Abbreviations:** sECG: Supine Electrocardiograms, paECG: Prone Anterior Electrocardiograms and pbECG: Prone Back Electrocardiograms.

**Supplementary Table 1b.-** QRS voltage change between supine and prone back lead placement in the healthy volunteer group.

| Patient | V1 | V2 | V3 | V4 | V5 | V6 |
| --- | --- | --- | --- | --- | --- | --- |
| 1 | -50.0% | -66.7% | -68.4% | -72.0% | -60.9% | 3.4% |
| 2 | -70.6% | -87.1% | -72.2% | -72.0% | -25.0% | -18.5% |
| 3 | -57.1% | -75.5% | -84.9% | -77.8% | -63.2% | 27.3% |
| 4 | -68.6% | -87.8% | -38.1% | -35.3% | -28.6% | 10.0% |
| 5 | -43.8% | -83.9% | -84.3% | -86.0% | -66.7% | -5.6% |
| 6 | -84.2% | -65.2% | -71.4% | -65.6% | -41.7% | 0.0% |
| 7 | -11.1% | -71.4% | -69.6% | -68.2% | -21.1% | -3.7% |
| 8 | -70.8% | -82.6% | -71.4% | -52.2% | -20.0% | 0.0% |
| 9 | -52.9% | -86.8% | -52.2% | -55.6% | -36.7% | 13.6% |
| 10 | -64.7% | -79.4% | -69.2% | -64.6% | -45.0% | -6.9% |
| 11 | -50.0% | -83.3% | -83.3% | -81.5% | -60.5% | -20.0% |
| 12 | -60.0% | -90.0% | -37.5% | -63.5% | -47.6% | 0.0% |
| 13 | -54.5% | -76.7% | -62.2% | -53.8% | -42.9% | -7.4% |
| 14 | -69.7% | -73.9% | -33.3% | -14.8% | -10.0% | 28.0% |
| 15 | -55.0% | -69.6% | -68.0% | -53.6% | -37.5% | 56.3% |
| 16 | -52.2% | -77.1% | -69.2% | -69.0% | -48.0% | 12.5% |
| 17 | -70.0% | -78.3% | -79.5% | -60.7% | 27.3% | -23.8% |
| 18 | -77.8% | -70.0% | -60.0% | -56.5% | -45.5% | 42.9% |
| 19 | -50.0% | -87.1% | -89.7% | -80.0% | -51.7% | 33.3% |
| 20 | -76.5% | -87.5% | -84.9% | -50.0% | -16.7% | 20.0% |
| 21 | -50.0% | -68.8% | -46.7% | -35.7% | -16.0% | 42.1% |
| 22 | -61.1% | -83.3% | -82.5% | -58.3% | -23.8% | 46.7% |
| 23 | -50.0% | -70.0% | -68.2% | -70.4% | -40.0% | 14.3% |
| 24 | -70.0% | -76.9% | -63.0% | -63.0% | -54.2% | -5.9% |
| 25 | -70.4% | -84.2% | -30.0% | -44.4% | -21.1% | 17.2% |
| 26 | -68.2% | -89.5% | -55.6% | -22.7% | -16.7% | -36.1% |
| 27 | -76.0% | -81.3% | -35.3% | -52.9% | -62.7% | -3.0% |
| 28 | -65.5% | -81.1% | -82.4% | -73.3% | -58.3% | 16.7% |
| 29 | -69.2% | -88.4% | -53.8% | -51.4% | -24.2% | 11.5% |
| 30 | -43.8% | -77.5% | -81.5% | -76.1% | -40.7% | -5.6% |
| 31 | -56.5% | -89.7% | -78.9% | -73.7% | -51.5% | 8.7% |
| 32 | 11.1% | -84.4% | -73.7% | -53.3% | -40.7% | 10.5% |
| 33 | -55.0% | -61.5% | -36.4% | -13.6% | -20.0% | 11.8% |
| 34 | -12.5% | -73.9% | -70.0% | -65.2% | -42.9% | 58.3% |
| 35 | -70.0% | -80.0% | -85.3% | -53.3% | -21.7% | -6.3% |
| 36 | -52.4% | -67.5% | -75.0% | -69.7% | -52.0% | 0.0% |
| 37 | -61.9% | -75.0% | -45.5% | -51.7% | -35.7% | 14.3% |
| 38 | -71.4% | -81.8% | -93.5% | -78.6% | -64.3% | 0.0% |
| 39 | -65.0% | -79.3% | -14.3% | -40.7% | -25.9% | 13.6% |
| 40 | -50.0% | -36.4% | -37.3% | -20.0% | -37.5% | 23.5% |

**Abbreviations:** sECG: Supine Electrocardiograms, paECG: Prone Anterior Electrocardiograms and pbECG: Prone Back Electrocardiograms.

**Supplementary Table 1c.-** QRS voltage change between supine and prone back lead placement in ARDS group**.**

| Patient | V1 | V2 | V3 | V4 | V5 | V6 |
| --- | --- | --- | --- | --- | --- | --- |
| 1 | -73.7% | -90.0% | -83.3% | -65.0% | -41.7% | 15.4% |
| 2 | -62.5% | -84.2% | -80.0% | -76.9% | -33.3% | -25.0% |
| 3 | -73.3% | -50.0% | -27.3% | -27.3% | -25.0% | 112.5% |
| 4 | -71.4% | -83.3% | -91.7% | -91.7% | -61.9% | 62.5% |
| 5 | 0.0% | -75.0% | -37.5% | -18.2% | -52.2% | 18.8% |
| 6 | -42.9% | -50.0% | -36.4% | -36.4% | -50.0% | 0.0% |
| 7 | -35.7% | -74.2% | -72.4% | -73.1% | 100.0% | -21.4% |
| 8 | -76.5% | -81.8% | -50.0% | -15.4% | -43.5% | 27.3% |
| 9 | -80.0% | -62.5% | -68.8% | -68.2% | -65.0% | -28.6% |
| 10 | -55.6% | -80.0% | -81.3% | -60.0% | -53.3% | 0.0% |
| 11 | -84.6% | -90.9% | -85.2% | -87.5% | -50.0% | -53.8% |
| 12 | -62.5% | -83.3% | -88.9% | -75.0% | -56.3% | 45.5% |
| 13 | -76.9% | -71.4% | -66.7% | -70.0% | -64.3% | -5.9% |
| 14 | -81.3% | -50.0% | -35.7% | -15.4% | -22.7% | 7.7% |
| 15 | -77.8% | -71.4% | 16.7% | 25.0% | -38.5% | 171.4% |
| 16 | -75.0% | -80.0% | -54.5% | -53.8% | -16.7% | -22.2% |
| 17 | -84.6% | -75.0% | -33.3% | 42.9% | -64.7% | 20.0% |
| 18 | -70.0% | -40.0% | -28.6% | -40.0% | 0.0% | -42.9% |
| 19 | -62.5% | -66.7% | -66.7% | -70.0% | -55.0% | 0.0% |
| 20 | -44.4% | -66.7% | -78.6% | -61.5% | -33.3% | -17.6% |
| 21 | -55.6% | -89.3% | -87.0% | -81.5% | -44.4% | -54.2% |
| 22 | -14.3% | -71.4% | -82.6% | -81.8% | -38.5% | 29.4% |
| 23 | -62.5% | -69.2% | -76.5% | -64.3% | 0.0% | -11.1% |
| 24 | -85.0% | -55.6% | 0.0% | 33.3% | -53.8% | 166.7% |
| 25 | -75.0% | -88.0% | -83.3% | -80.0% | -9.1% | -15.8% |
| 26 | -62.5% | -71.4% | -80.0% | -57.1% | -52.9% | 0.0% |
| 27 | -81.8% | -81.8% | -80.0% | -77.8% | -32.0% | 71.4% |
| 28 | -75.0% | -57.1% | -44.4% | -44.4% | -7.1% | -44.4% |
| 29 | 0.0% | -57.1% | -81.0% | -73.7% | -41.7% | -11.1% |
| 30 | -76.9% | -88.0% | -81.8% | -76.2% | -33.3% | -33.3% |
| 31 | -55.6% | -65.0% | -71.0% | -42.9% | -25.0% | 27.8% |
| 32 | 0.0% | -80.0% | -77.8% | -62.5% | -61.9% | 22.2% |
| 33 | -81.8% | -93.3% | -81.8% | -33.3% | -52.2% | 20.0% |
| 34 | -40.0% | -72.7% | -87.0% | -88.9% | -50.0% | 100.0% |
| 35 | -75.0% | -75.0% | -69.2% | -38.5% | 100.0% | 21.4% |
| 36 | -71.4% | -85.7% | -87.0% | -80.0% | -43.5% | -7.7% |
| 37 | -54.5% | -82.6% | -70.6% | -68.2% | -65.0% | 8.3% |
| 38 | -62.5% | -50.0% | -91.7% | -86.7% | -53.3% | 28.6% |
| 39 | -60.0% | -83.3% | -88.9% | -75.0% | -50.0% | 75.0% |
| 40 | -75.0% | -84.2% | -76.5% | -54.5% | -56.3% | 40.0% |
| 41 | -57.1% | -83.3% | -80.0% | -73.3% | -64.3% | -10.0% |
| 42 | -62.5% | -60.0% | -40.0% | -18.2% | -22.7% | 0.0% |
| 43 | -50.0% | -66.7% | -73.3% | -70.6% | -38.5% | -8.3% |
| 44 | -50.0% | -63.6% | -66.7% | -60.0% | -16.7% | 27.8% |
| 45 | -84.6% | -61.5% | 14.3% | -16.7% | -64.7% | 8.3% |

**Abbreviations:** sECG: Supine Electrocardiograms, paECG: Prone Anterior Electrocardiograms and pbECG: Prone Back Electrocardiograms.

**Supplementary Table 2a.-** Comparison of QRS morphology in the supine, prone anterior, and prone back positions in healthy volunteers. No comparison was made in the V6 position since lead placement position was the same for sECG, paECG and pbECG.

| PATIENT | V1 SUPINE | V1 PRONE ANTERIOR | V1 PRONE BACK | V2 SUPINE | V2 PRONE ANTERIOR | V2 PRONE BACK | V3 SUPINE | V3 PRONE ANTERIOR | V3 PRONE BACK | V4 SUPINE | V4 PRONE ANTERIOR | V4 PRONE BACK | V5 SUPINE | V5 PRONE ANTERIOR | V5 PRONE BACK | |
| --- | --- | --- | --- | --- | --- | --- | --- | --- | --- | --- | --- | --- | --- | --- | --- | --- |
| 1 | rS | rS | Qr | RS | rS | qR | RS | RS | qR | Rs | Rs | qR | Rs | Rs | qR |  |
| 2 | rS | rS | Qr | rS | rS | Qr | RS | RS | qR | qRs | qRs | qR | qR | qRs | qR |  |
| 3 | rS | rs | qR | rS | rS | qR | rS | rS | qR | RS | RS | R | Rs | Rs | Rs |  |
| 4 | rS | rS | Qr | rS | rS | Qr | RS | rS | qR | Rs | Rs | qR | Rs | Rs | qR |  |
| 5 | rS | rsr' | qR | rS | rS | qR | rS | rS | qR | RS | RS | qR | Rs | RS | Rs |  |
| 6 | rS | rS | Qr | rS | rS | Qr | RS | RS | qR | Rs | Rs | qR | Rs | Rs | R |  |
| 7 | rS | rS | Qr | rS | Rs | QR | Rs | Rs | qR | Rs | Rs | qR | Rs | Rs | Rs |  |
| 8 | rS | Rs | Qr | rS | rS | qR | Rs | RS | qR | Rs | Rs | qR | R | Rs | R |  |
| 9 | rS | rS | Qr | rS | rS | Qr | RS | RS | qR | qRs | Rs | qR | qR | qRs | qR |  |
| 10 | rS | rS | Qr | rS | rS | qR | RS | RS | qR | Rs | RS | qR | qRs | Rs | qR |  |
| 11 | rS | rS | qR | rS | rS | qR | RS | rS | qR | RS | RS | qR | Rs | Rs | Rs |  |
| 12 | rS | rS | Qr | rS | rS | Qr | Rs | rS | qR | Rs | Rs | qR | qRs | Rs | qR |  |
| 13 | rS | rS | Qr | rS | rS | qR | Rs | RS | qR | Rs | Rs | qR | R | Rs | qR |  |
| 14 | rS | rS | QR | rS | rS | qR | rS | rS | qR | Rs | RS | qR | Rs | Rs | R |  |
| 15 | rS | rS | QR | rS | rSr' | qR | rS | rS | R | RS | RS | R | Rs | Rs | Rs |  |
| 16 | rS | rS | QR | rS | rS | qR | RS | RS | qR | Rs | Rs | qR | Rs | Rs | Rs |  |
| 17 | rS | rSr' | Qr | rS | rS | qR | rS | rS | qR | Rs | RS | qR | qR | Rs | qR |  |
| 18 | rS | rS | Qr | rS | rS | Qr | rS | rS | qR | Rs | Rs | Rs | Rs | Rs | Rs |  |
| 19 | rS | rS | qR | rS | rS | Qr | RS | rS | Qr | Rs | RS | R | Rs | Rs | Rs |  |
| 20 | rS | rS | Qr | rS | rS | Qr | RS | rS | qR | Rs | Rs | qR | qRs | Rs | qR |  |
| 21 | rS | rS | Qr | rS | rS | qR | RS | rS | qR | Rs | RS | qR | Rs | Rs | qRs |  |
| 22 | rS | rS | qR | rS | rS | qR | rS | rS | qR | rS | rS | qR | Rs | Rs | qRs |  |
| 23 | rS | rS | QR | rS | rS | qR | rS | rS | qR | Rs | Rs | qR | Rs | Rs | Rs |  |
| 24 | rS | rS | qR | rS | rS | qR | rS | rS | qR | RS | Rs | qR | Rs | Rs | qR |  |
| 25 | rS | rS | QR | rS | rS | qR | rS | rS | qR | Rs | Rs | qR | Rs | Rs | qR |  |
| 26 | rSr´ | rSr' | QR | rS | rS | qR | rS | rS | qR | Rs | Rs | qR | qRs | Rs | Rs |  |
| 27 | rS | rS | Qr | rS | rS | qR | rS | rS | qR | Rs | Rs | qR | qR | Rs | qR |  |
| 28 | rS | rS | qR | rS | rS | qR | rS | rS | qR | RS | RS | R | Rs | Rs | R |  |
| 29 | rS | rS | Qr | rS | rS | qR | rS | rS | qR | Rs | Rs | qR | qRs | qRs | qR |  |
| 30 | rS | rSr' | QR | rS | rS | qR | rS | rS | qR | Rs | Rs | Rs | Rs | Rs | Rs |  |
| 31 | rS | rS | Qr | RS | RS | Qr | Rs | RS | qR | Rs | Rs | qR | Rs | Rs | R |  |
| 32 | rSr's' | rSR' | Qr | RS | rS | qR | Rs | Rs | qR | Rs | Rs | qRs | Rs | Rs | Rs |  |
| 33 | rS | rS | Qr | rS | rS | Qr | RS | RS | qR | Rs | Rs | qR | R | Rs | qR |  |
| 34 | rS | rS | qr | rS | rS | Qr | RS | RS | qR | Rs | Rs | R | Rs | Rs | Rs |  |
| 35 | rS | rS | Qr | rS | rS | qR | rS | rS | qR | rS | rS | qR | Rs | Rs | R |  |
| 36 | rS | rS | Qr | rS | rS | qR | RS | RS | qR | Rs | Rs | qR | Rs | Rs | R |  |
| 37 | rS | rS | QR | rS | rS | qR | rS | rS | R | Rs | Rs | R | Rs | Rs | R |  |
| 38 | rS | rSr' | q | rSr' | rSr' | Qr | rS | rS | qrs | RS | rS | qR | Rs | Rs | Rs |  |
| 39 | rS | rS | Q | rS | rS | Qr | Rs | RS | qR | Rs | Rs | qR | qR | R | qR |  |
| 40 | rSr' | rsr' | qr | rsr' | rSr' | qR | rSr' | rSr' | qR | rS | rS | qR | Rs | Rs | qRs |  |

**Abbreviations:** sECG: Supine Electrocardiograms, paECG: Prone Anterior Electrocardiograms and pbECG: Prone Back Electrocardiograms.

**Supplementary Table 2b.-** Comparison of QRS morphology in the supine and prone back positions in ARDS patients. No comparison was made in the V6 position since lead placement position was the same for sECG, paECG and pbECG.

| PATIENT | V1 SUPINE | V1 PRONE BACK | V2 SUPINE | V2 PRONE BACK | V3 SUPINE | V3 PRONE BACK | V4 SUPINE | V4 PRONE BACK | V5 SUPINE | V5 PRONE BACK |
| --- | --- | --- | --- | --- | --- | --- | --- | --- | --- | --- |
| 1 | rS | QS | rS | Qr | RS | qR | Rs | qR | qR | qR |
| 2 | rS | QR | rS | qR | RS | qR | Rs | qR | Rs | qR |
| 3 | rS | Qr | rS | qR | RS | qR | Rs | qR | R | qR |
| 4 | QS | Unreadable | rS | Unreadable | Rs | unreadable | Rs | R | Rs | Rs |
| 5 | QS | Unreadable | rS | QR | rS | qR | Rs | qR | Rs | qR |
| 6 | rS | Qr | RS | qR | Rs | qR | Rs | qR | Rs | qR |
| 7 | rS | QR | rS | qR | rS | qR | rS | qR | RS | qR |
| 8 | rS | Qr | rS | qR | RS | qR | Rs | qR | Rsr' | qR |
| 9 | rSr' | Unreadable | qRs | qR | qRs | qR | qR | qR | qR | qR |
| 10 | rS | QS | rS | Unreadable | qR | R | R | R | R | Rs |
| 11 | rS | qR | rS | qR | rS | qR | rS | qR | rS | qR |
| 12 | QS | Qr | rS | qR | Rs | R | Rs | R | Rs | qR |
| 13 | rS | QS | RS | Unreadable | Rsr' | qR | R | qR | qR | qR |
| 14 | rsR' | QR | rsR' | qRs | rsR' | qRs | Rsr' | qRs | Rs | qRs |
| 15 | rSr' | QR | rSr' | qR | Rs | qR | Rs | qR | Rs | qRs |
| 16 | rS | QS | rS | R | Rs | qR | Rs | qR | R | qR |
| 17 | rS | Qr | rS | qR | rS | qR | Rs | qR | R | qR |
| 18 | rS | Qr | Rs | qR | Rs | qR | Rs | R | R | R |
| 19 | rS | QR | rS | qR | rS | qR | Rs | qR | Rs | qR |
| 20 | qR | Qr | Rsr' | Unreadable | Rsr' | unreadable | Rs | Rs | Rs | Rs |
| 21 | rS | Qr | rS | qR | rS | qR | rS | qR | Rs | qR |
| 22 | rS | Qr | rS | QR | Rs | qR | qRs | qR | qRs | qR |
| 23 | QS | qR | rS | qR | rS | R | rS | R | rS | R |
| 24 | rS | Qr | rS | qR | qR | qR | qR | qR | qR | qR |
| 25 | rS | Qr | rS | qR | rS | R | Rs | R | Rs | R |
| 26 | rS | Qr | Rs | qR | Rs | qR | Rs | R | Rs | R |
| 27 | rS | qR | rS | qR | rS | qR | rS | R | RS | RS |
| 28 | QS | QR | rS | qR | Rs | R | Rs | R | Rs | R |
| 29 | QS | QR | rS | qR | rS | qR | Rs | qR | Rs | R |
| 30 | rS | qR | rS | qR | rS | R | rS | R | Rs | R |
| 31 | rS | qR | rS | qR | rS | qR | rS | qR | qRs | qR |
| 32 | QS | Qr | RS | qR | RS | R | Rs | Rs | Rs | Rs |
| 33 | rS | Qr | RS | Unreadable | Rs | R | R | R | qR | R |
| 34 | QS | qR | rS | qR | rS | R | rS | R | Rs | R |
| 35 | rS | Qr | rS | qR | Rs | qR | Rs | qR | R | qR |
| 36 | rS | Qr | rS | QR | rS | qR | Rs | qR | qRs | qR |
| 37 | rS | Qr | rS | QR | rS | qR | qRs | qR | qRs | qRs |
| 38 | Paced | paced | Paced | Paced | Paced | paced | paced | paced | paced | paced |
| 39 | rS | QR | rS | qR | Rs | qR | Rs | qR | qR | qR |
| 40 | QS | Qr | rS | Unreadable | rS | unreadable | Rs | Rs | Rs | Rs |
| 41 | rS | Qr | rS | QR | Rs | qR | Rs | qR | Rsr' | qR |
| 42 | rS | QR | rS | qR | rS | qR | Rs | qR | qR | qR |
| 43 | rS | QR | rS | qR | rS | qR | Rs | qR | Rs | qR |
| 44 | rSr' | qR | Rsr' | qR | rS | qRs | RS | qRs | Rs | qRs |
| 45 | rS | Unreadable | rS | R | R | R | R | R | R | R |

**Abbreviations:** sECG: Supine Electrocardiograms, paECG: Prone Anterior Electrocardiograms and pbECG: Prone Back Electrocardiograms.

**Supplementary Table 3a.-** T wave morphology in the healthy volunteer group in the supine, prone anterior, and prone back positions. No comparison was made in the V6 position since lead placement position was the same for sECG, paECG and pbECG.

| PATIENT | V1 SUPINE | V1 PRONE ANTERIOR | V1 PRONE BACK | V2 SUPINE | V2 PRONE ANTERIOR | V2 PRONE BACK | V3 SUPINE | V3 PRONE ANTERIOR | V3 PRONE BACK | V4 SUPINE | V4 PRONE ANTERIOR | V4 PRONE BACK | V5 SUPINE | V5 PRONE ANTERIOR | V5 PRONE BACK |
| --- | --- | --- | --- | --- | --- | --- | --- | --- | --- | --- | --- | --- | --- | --- | --- |
| 1 | FLAT | - | - | + | + | FLAT | + | + | + | + | + | + | + | + | + |
| 2 | - | - | - | + | + | FLAT | + | + | + | + | + | + | + | + | + |
| 3 | + | - | - | + | + | FLAT | + | + | + | + | + | + | + | + | + |
| 4 | - | - | - | + | + | FLAT | + | + | + | + | + | + | + | + | + |
| 5 | + | - | - | + | + | FLAT | + | + | FLAT | + | + | + | + | + | + |
| 6 | + | + | - | + | + | FLAT | + | + | + | + | + | + | + | + | + |
| 7 | - | - | - | + | + | - | + | + | + | + | + | + | + | + | + |
| 8 | + | + | - | + | + | - | + | + | FLAT | + | + | + | + | + | + |
| 9 | - | - | - | + | + | FLAT | + | + | + | + | + | + | + | + | + |
| 10 | - | - | - | + | + | FLAT | + | + | + | + | + | + | + | + | + |
| 11 | + | + | - | + | + | UNREADABLE | + | + | UNREADABLE | + | + | + | + | + | + |
| 12 | - | - | - | + | + | - | + | + | + | + | + | + | + | + | + |
| 13 | FLAT | - | - | + | + | FLAT | + | + | + | + | + | + | + | + | + |
| 14 | - | - | - | - | FLAT | FLAT | - | + | + | + | + | + | + | + | + |
| 15 | - | - | - | + | FLAT | FLAT | + | + | + | + | + | + | + | + | + |
| 16 | - | - | FLAT | FLAT | FLAT | + | + | FLAT | + | + | + | + | + | + | + |
| 17 | - | - | - | + | - | FLAT | + | + | + | + | + | + | + | + | + |
| 18 | + | - | FLAT | + | + | - | + | + | + | + | + | + | + | + | + |
| 19 | FLAT | FLAT | - | + | + | - | + | + | FLAT | + | + | + | + | + | + |
| 20 | - | - | - | + | + | - | + | + | + | + | + | + | + | + | + |
| 21 | - | - | - | + | - | + | + | + | + | + | + | + | + | + | + |
| 22 | - | - | - | + | + | FLAT | + | + | + | + | + | + | + | + | + |
| 23 | - | - | FLAT | + | + | FLAT | - | +/- | + | + | + | + | + | + | + |
| 24 | - | - | - | + | FLAT | FLAT | FLAT | + | + | + | + | + | + | + | + |
| 25 | - | - | - | + | + | - | + | + | + | + | + | + | + | + | + |
| 26 | - | - | - | - | flat | FLAT | + | + | + | + | + | + | + | + | + |
| 27 | + | - | - | + | + | - | + | + | + | + | + | + | + | + | + |
| 28 | + | + | - | + | + | - | + | + | FLAT | + | + | + | + | + | + |
| 29 | - | - | - | + | + | FLAT | + | + | + | + | + | + | + | + | + |
| 30 | - | - | - | + | + | FLAT | + | + | + | + | + | + | + | + | + |
| 31 | + | - | - | + | + | FLAT | + | + | FLAT | + | + | + | + | + | + |
| 32 | - | - | - | + | + | FLAT | + | + | + | + | + | + | + | + | + |
| 33 | + | + | - | + | + | - | + | + | + | + | + | + | + | + | + |
| 34 | - | - | FLAT | + | + | FLAT | + | + | + | + | + | + | + | + | + |
| 35 | - | - | - | + | - | FLAT | + | + | FLAT | + | + | + | + | + | + |
| 36 | - | - | - | + | + | FLAT | + | + | + | + | + | + | + | + | + |
| 37 | FLAT | - | - | + | + | FLAT | + | + | + | + | + | + | + | + | + |
| 38 | - | - | - | - | - | FLAT | + | + | + | + | + | + | + | + | + |
| 39 | - | - | - | + | + | FLAT | + | + | + | + | + | + | + | + | + |
| 40 | - | - | - | + | - | + | FLAT | - | + | FLAT | flat | + | + | + | + |

**Abbreviations:** sECG: Supine Electrocardiograms, paECG: Prone Anterior Electrocardiograms and pbECG: Prone Back Electrocardiograms.

**Supplementary Table 3b.-** T wave morphology in ARDS group in supine and prone back positions. No comparison was made in the V6 position since lead placement position was the same for sECG, paECG and pbECG.

| PATIENT | V1 SUPINE | V1 PRONE BACK | V2 SUPINE | V2 PRONE BACK | V3 SUPINE | V3 PRONE BACK | V4 SUPINE | V4 PRONE BACK | V5 SUPINE | V5 PRONE BACK | V6 SUPINE | V6 PRONE BACK |
| --- | --- | --- | --- | --- | --- | --- | --- | --- | --- | --- | --- | --- |
| 1 | + | - | + | - | + | flat | + | flat | + | + | + | + |
| 2 | flat | flat | + | flat | + | flat | + | flat | + | flat | + | flat |
| 3 | flat | flat | + | flat | flat | flat | + | flat | + | + | + | + |
| 4 | + | - | + | - | + | - | + | - | + | - | flat | + |
| 5 | - | unreadable | + | flat | + | + | + | + | + | + | + | + |
| 6 | - | flat | + | flat | + | flat | + | flat | + | flat | + | flat |
| 7 | + | + | + | flat | + | - | flat | - | - | - | - | - |
| 8 | - | - | + | flat | + | + | + | + | + | + | + | + |
| 9 | - | - | + | flat | + | flat | + | flat | + | + | + | + |
| 10 | flat | - | + | flat | + | flat | + | + | + | + | + | + |
| 11 | + | flat | + | flat | + | flat | + | flat | + | flat | + | flat |
| 12 | flat | - | + | flat | biphasic | flat | biphasic | flat | + | + | + | + |
| 13 | flat | flat | + | flat | + | flat | + | flat | + | + | + | + |
| 14 | - | flat | - | flat | - | + | + | + | + | + | + | + |
| 15 | - | flat | + | flat | + | flat | + | + | + | + | + | + |
| 16 | flat | flat | + | flat | + | flat | + | flat | + | + | + | + |
| 17 | flat | flat | + | flat | + | flat | + | + | + | + | + | + |
| 18 | unreadable | flat | + | flat | + | + | + | + | + | + | + | + |
| 19 | - | - | + | flat | + | + | + | + | + | + | + | + |
| 20 | - | flat | + | flat | + | + | + | + | + | + | + | + |
| 21 | + | + | + | flat | + | - | - | - | - | - | - | - |
| 22 | flat | - | + | flat | + | flat | + | + | + | + | + | + |
| 23 | + | flat | + | flat | + | flat | + | flat | biphasic | - | biphasic | - |
| 24 | + | flat | + | flat | - | flat | - | - | - | - | - | - |
| 25 | + | flat | + | flat | + | flat | biphasic | - | - | - | - | - |
| 26 | + | - | + | unreadable | + | flat | + | flat | + | + | + | + |
| 27 | + | - | + | - | + | - | + | flat | + | + | + | + |
| 28 | + | flat | + | flat | + | unreadable | + | + | + | + | + | + |
| 29 | - | flat | + | flat | + | + | + | + | + | + | + | + |
| 30 | + | - | + | - | + | - | + | + | + | + | + | + |
| 31 | flat | flat | + | unreadable | + | unreadable | + | + | + | + | + | + |
| 32 | + | - | + | flat | + | + | + | + | + | + | + | + |
| 33 | - | - | flat | - | + | flat | + | flat | + | + | + | + |
| 34 | + | - | + | - | + | - | + | flat | + | + | + | + |
| 35 | flat | - | + | flat | + | flat | + | + | + | + | + | + |
| 36 | - | flat | flat | flat | + | unreadable | + | + | + | + | + | + |
| 37 | + | - | + | - | + | flat | + | + | + | + | + | + |
| 38 | + | - | + | - | + | flat | + | + | + | + | + | + |
| 39 | + | - | + | flat | + | flat | + | + | + | + | + | + |
| 40 | - | - | + | flat | + | flat | + | flat | + | + | + | + |
| 41 | - | - | + | - | + | flat | + | + | + | + | + | + |
| 42 | - | - | + | flat | + | + | + | + | + | + | + | + |
| 43 | flat | flat | + | flat | + | flat | + | + | + | + | + | + |
| 44 | + | flat | + | flat | + | - | + | - | - | - | - | - |
| 45 | + | - | + | flat | + | + | + | + | + | + | + | + |

**Abbreviations:** sECGs: Supine Electrocardiograms, paECGs: Prone Anterior Electrocardiograms and pbECG: Prone Back Electrocardiograms.

**Supplementary Table 4:** CT measurements in the supine and prone positions

| **Patient** | **Apex to anterior ribs/chest wall (mm)** | | **Apex to posterior ribs/chest wall (mm)** | | **Pericardial surface in contact with anterior chest wall (mm)** | |
| --- | --- | --- | --- | --- | --- | --- |
|  | **Supine** | **Prone** | **Supine** | **Prone** | **Supine** | **Prone** |
| **1** | 25.1 | 17.1 | 146.5 | 138.2 | 90.4 | 104.1 |
| **2** | 14.8 | 16.5 | 151 | 149.2 | 32.4 | 104.3 |
| **3** | 20.3 | 11.3 | 146.4 | 138.8 | 43.3 | 95.1 |
| **4** | 8.5 | 10.5 | 131.2 | 123.5 | 79.9 | 95.3 |
| **5** | 14.2 | 7.4 | 142.1 | 133.5 | 82.4 | 136.6 |
| **6** | 19 | 13.3 | 123.1 | 147.7 | 95.6 | 131.7 |
| **7** | 22.5 | 11 | 174.1 | 164.8 | 45.5 | 97.3 |
| **8** | 20.2 | 6.7 | 122.1 | 122.8 | 38 | 99 |
| Mean | **18** | **11.7** | **142** | **139.8** | **63.4** | **107.9** |

**Abbreviations:** CT: computed tomography.

| **Supplementary Table 5: Univariate analysis of ECG findings on supine and prone ECGs for ARDS positive patients (n = 45)** | | | |
| --- | --- | --- | --- |
| ECG Characteristic (mean ± SD unless specified) | Prone back | Supine | p-value (t-test) |
| Heart rate | 85.3 ± 17.2 | 85.4 ± 16.9 | p = 0.973 |
| PR interval (ms) | 158.0 ± 24.0 | 157.8 ± 20.9 | p = 0.249 |
| QRS duration (ms) | 84.2 ± 22.7 | 92.7 ± 19.2 | **p < 0.0001** |
| QTc (Bazzett, ms) | 436.0 ± 32.5 | 451.5 ± 33.2 | p = 0.010 |
| Lead V1 amplitude (mV) | 0.34 ± 0.13 | 1.08 ± 0.51 | **p < 0.0001** |
| Lead V2 amplitude (mV) | 0.33 ± 0.15 | 1.38 ± 0.71 | **p < 0.0001** |
| Lead V3 amplitude (mV) | 0.46 ± 0.21 | 1.60 ± 0.67 | **p < 0.0001** |
| Lead V4 amplitude (mV) | 0.62 ± 0.26 | 1.65 ± 0.75 | **p < 0.0001** |
| Lead V5 amplitude (mV) | 1.04 ± 0.84 | 1.42 ± 0.50 | **p = 0.013** |
| Lead V6 amplitude (mV) | 1.23 ± 0.48 | 1.14 ± 0.47 | p = 0.237 |
| QRS axis (deg) | 7.1 ± 50.3 | 9.8 ± 49.0 | p = 0.594 |

Abbreviations: SD: standard deviation, Ms: millisecond, mV: millivolts Deg: degree

Data from the ARDS group showing the difference between the prone back and supine ECG parameters. Text in bold identifies significant parameters.
