# Supplementary figures and images for: Anatomical considerations and clinical interpretation of the 12-lead ECG in the prone position: a prospective multicentre study
Source: Europace. 2022 Oct 5;25(1):175–84. doi: 10.1093/europace/euac099 (PMC10103558; doi:10.1093/europace/euac099)

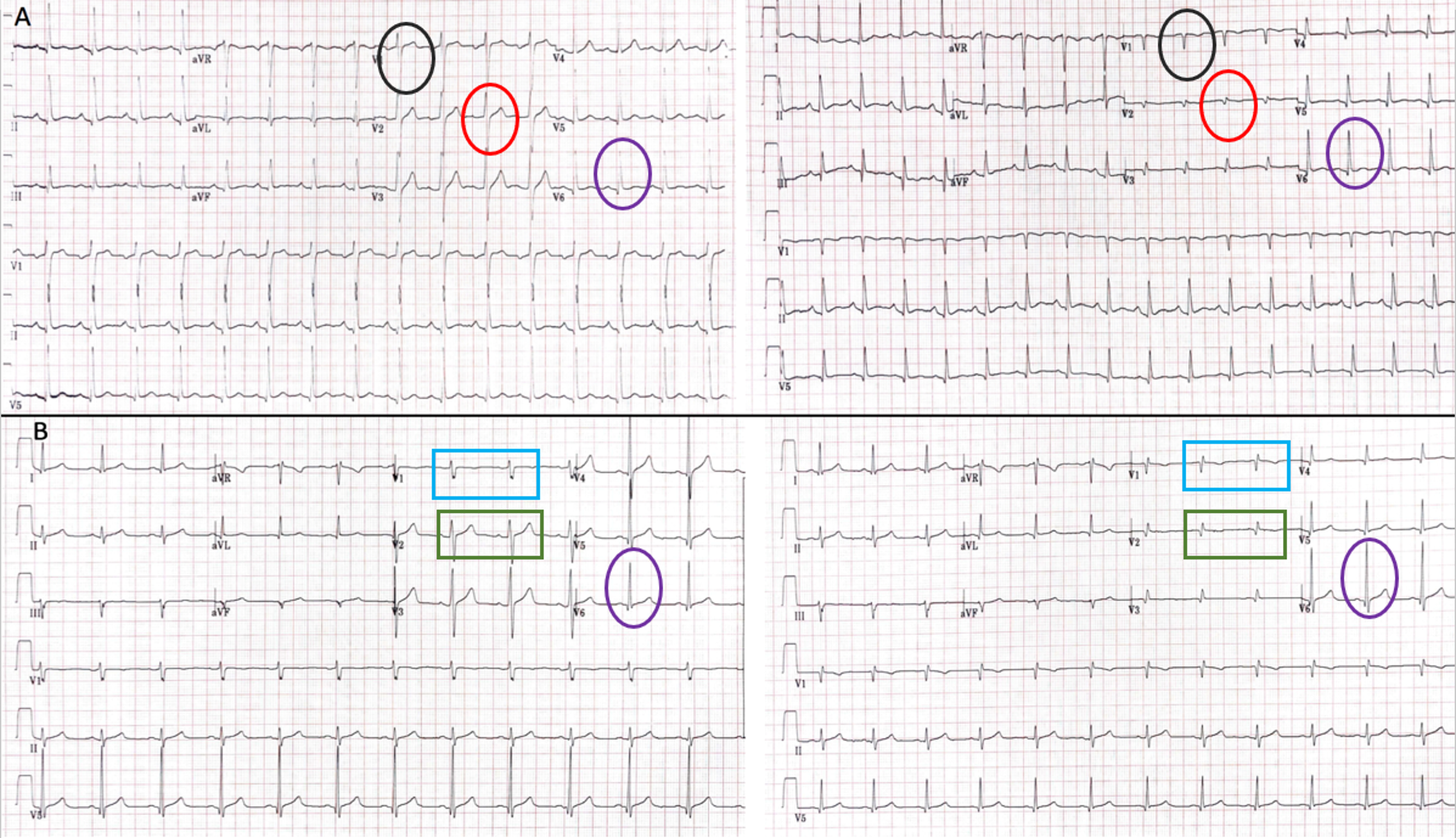

Supplement: euac099_Supplementary_Data [file euac099_supplementary_data.zip › Prone ECG Supplementary material - Figure 1.tif]
